# Update on the Epidemiology of Macrolide-Resistant *Mycoplasma pneumoniae* in Europe: A Systematic Review

**DOI:** 10.3390/idr13030073

**Published:** 2021-09-02

**Authors:** Daniela Loconsole, Anna Lisa De Robertis, Anna Sallustio, Francesca Centrone, Caterina Morcavallo, Silvia Campanella, Marisa Accogli, Maria Chironna

**Affiliations:** 1 Department of Biomedical Sciences and Human Oncology-Hygiene Section, University of Bari, 70124 Bari, Italy; daniela.loconsole@uniba.it (D.L.); derobertis.annalisa@gmail.com (A.L.D.R.); francesca.centrone.fc@gmail.com (F.C.); caterinamorcavallo@gmail.com (C.M.); campanella.silvia@libero.it (S.C.); accoisa@gmail.com (M.A.); 2 Hygiene Unit, Azienda Ospedaliero-Universitaria Consorziale Policlinico di Bari, 70124 Bari, Italy; annasallustio@libero.it

**Keywords:** epidemiology, macrolide-resistant *Mycoplasma pneumoniae*, Europe, antimicrobial resistance, systematic review

## Abstract

Macrolide-resistant *Mycoplasma pneumoniae* (MR-MP) infections cause upper and lower respiratory tract infections in both children and adults, and are characterized by a longer duration of symptoms. Here, we undertook a systematic review of studies on MR-MP in Europe. The review meets PRISMA guidelines. The PubMed, Scopus, and Science Direct databases were searched using suitable keywords to identify relevant studies published from 2010 to 2021; 21 studies were included. Overall, a low level of MR-MP spread was reported in Europe. MR-MP spread increased during epidemic waves registered in Europe, particularly in Italy and Scotland, where the highest MR-MP infection rates were registered during the 2010–2011 epidemic. By contrast, no MR-MP infections were reported in Finland and the Netherlands. Continued monitoring of MR-MP in Europe is needed to maintain the low rates of infection. Moreover, a coordinated and structured pan-European surveillance program adequate for public health surveillance is advisable, with the purpose of containing the spread of antimicrobial resistance.

## 1. Introduction

*Mycoplasma pneumoniae* (MP) is a common cause of upper and lower respiratory tract disease in both children and adults. In the post-pneumococcal conjugate vaccine (PCV) 13 era, MP has become the leading cause of pediatric community-acquired pneumonia (CAP) in countries where PCV13 is included in the national immunization program [1,2]. Clinical symptoms are usually mild, although occasionally MP infection can be life-threatening as it can cause extra-respiratory manifestations, mainly skin lesions, hematologic disorders, and cardiovascular and nervous disease [3,4,5]. The pathogenesis of such extrapulmonary manifestations is more likely due to the host immune response rather than to the pathogen itself [6]. MP infections are both endemic and epidemic worldwide. Epidemics occur at intervals of 4–7 years [7,8,9]. It is hypothesized that these fluctuations are related to antigenic shifts in strains, decreased herd immunity in populations, or both [7,10]. During such periods, MP is responsible for 25% of all CAP cases [8], while the incidence drops to 1–8% between epidemics [11].

The majority of MP infections are self-limiting, especially in adults. Beta-lactam antimicrobial drugs are ineffective against MP since the pathogen lacks a cell wall. Macrolides, tetracyclines, and fluoroquinolones are the first-choice treatments, but only macrolides are recommended for children due to the age-related adverse effects of tetracyclines and fluoroquinolones [4,12]. However, the use of tosufloxacin has been approved in Japan for pediatric patients since 2010, and recent data showed that no changes in MP susceptibility to this antibiotic have been reported [13]. Inappropriate use, or overuse, of macrolides has led to worldwide reporting of macrolide-resistant MP strains (MR-MP). MR-MP were first isolated from pediatric patients with CAP in 2001 [14]. Since then, the number of resistant strains has increased rapidly year by year [15]. Macrolide resistance phenotypes of MP, which are defined by single nucleotide polymorphisms in the V domain of the single-copy 23S rRNA gene [15,16], are more common in children than in adults [17]. The mutations that induce a high-level of macrolide resistance include the transition A2063G and the transversion A2064G, whereas the A2617G transition confers low-level resistance [15]. Although macrolide resistance is mainly due to point mutations in domain V of the 23S rRNA gene, recent studies show that the macrolide efflux pump may contribute to macrolide resistance [18]. The clinical manifestations of MP infection are similar, regardless of macrolide resistance [19]. However, MR-MP infections lead to a longer duration of fever, cough, hospital stay, and antibiotic administration [20]. MP infection can lead to prolonged carriage; therefore, infected people represent a reservoir for spread to other people [21]. In addition, asymptomatic carriage of MR-MP has been described [22]. Macrolide resistance rates range from 0.2% in Sweden [23] to >90% in China [24]. The present review was conducted to examine the spread of MR-MP in Europe from 2010 to the present day to gain insight into the emergence of resistant strains in European countries in light of the lack of specific surveillance systems.

## 2. Materials and Methods

A systematic review of all peer-reviewed studies concerning MR-MP infections in Europe from 2010 to the present day was performed according to the Preferred Reporting Items for Systematic Reviews and Meta-Analyses (PRISMA) guidelines. First, the PubMed, Scopus, and Science Direct databases were searched to identify relevant literature. Data extraction was performed from January 2010 to June 2021 using the following keywords: “(Mycoplasma pneumoniae) AND (macrolide) AND (resistance) AND (Europe)”. The screening of resources took place during the first phase by reading the title and abstract of the studies, followed by removal of duplicates. During the second stage, articles were considered eligible if they met the following inclusion criteria: English language studies, case reports or research articles, and a focus on MP infection. Book chapters, reviews, conference abstracts, practice guidelines, non-European studies, and studies of other pathogens were excluded.

## 3. Results

**Figure 1:**
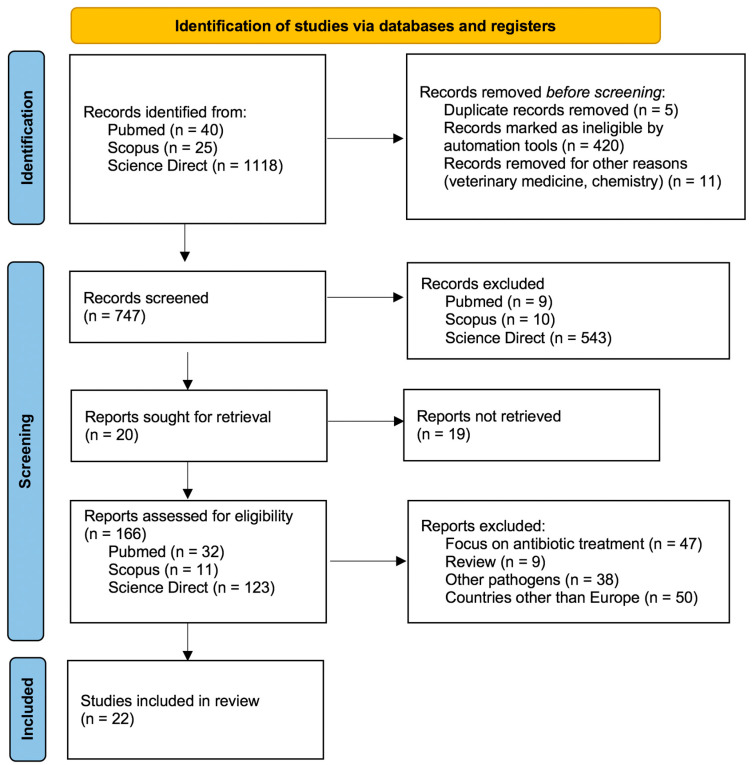
PRISMA flow chart.

The initial search identified 1183 articles (Figure 1). 

After removing duplicates and studies marked as ineligible by automation tools, 747 articles were screened by title and abstract. Irrelevant articles were excluded, and 20 articles were retrieved for full-text assessment. Of these, only one was suitable. Articles that focused on antibiotic treatments, studies conducted in countries other than in Europe or on pathogens other than MP, and reviews were excluded. Finally, 22 studies were included in the systematic review (Table 1).

In Germany, Dumke et al. reported that the prevalence of MR-MP infections among children and adults during the years 2009–2012 was 3.6%. The study population included both outpatients and inpatients [25]. Moreover, a prospective study performed on 783 CAP adult patients in the 2011–2012 period showed a 3.1% MR-MP infection rate among 96 MP positive patients [26]. The reported prevalence of MR-MP remained stable (at 3.0%) from 2016 to 2018 [27]. A pediatric case of macrolide resistance after macrolide treatment was reported in December 2013 [28]. A pharyngeal swab collected from a 15-year-old patient taken on the first day of hospitalization was positive for a macrolide-susceptible MP strain. On Day 19 from symptom onset, the patient required a new hospital admission due to multiple severe skin lesions. The pharyngeal swab collected during the second hospitalization harbored both wild-type MP sequences and clones carrying two mutations typical of a macrolide-resistant strain, suggesting that the MR-MP mutants were selected from the wild-type MP strain [28]. 

In Italy, the first identification of MR-MP infection in children dates back to 2010 [16], while in adults it was first described during a familial outbreak in 2015 [22]. The prevalence of MR-MP in children during the 2010 epidemic in Italy was estimated to be 26% [16], whereas a study in 2019 reported a prevalence of 1.3% among adults with CAP [29]. In 2013, Cardinale et al. demonstrated that the presence of an MR-MP infection in children does not change the presentation of CAP compared with that of macrolide-susceptible MP infection [19]. However, children with MR-MP infection showed a longer duration of fever, cough, hospitalization, and antibiotic administration [19]. 

The reported prevalence of MR-MP in France in the 2007–2010 endemic period was 3.4% [30]. In 2011, when an increased incidence of MP infection was registered in most European countries, macrolide resistance-associated mutations were found in samples collected in Bordeaux and Caen, raising the prevalence to 8.3% in both children and adults [31].

In Spain, MR-MP infection was first identified in 2012 in a 23-year-old Chinese female who returned from China 13 days before CAP onset [32]. After the diagnosis of MP infection, the patient was first treated with macrolides until her clinical condition worsened. Due to the high-rate of macrolide resistance in China, antibiotic treatment was changed to doxycycline, which resulted in a rapid improvement in clinical status. Moreover, in Spain, a retrospective analysis of MP infections in children and adults during 2013–2017 revealed that the prevalence of MR-MP strains was 8% [33]. 

In the UK, a single-center laboratory-based analysis in Scotland reported MR-MP detection rates of 19% during the epidemic of 2010–2011 [34]. In England and Wales, the rate of macrolide resistance was 9.3% in the 2014–2015 period [35]. 

In Slovenia, the occurrence of MR-MP infections seems sporadic. During a period covering two nationwide epidemics (2010–2011 and 2013–2014), circulation of MR-MP remained low. In particular, a retrospective study of both adults and children during 2006–2014 reported MR-MP infections in 1% of cases [36], and macrolide resistance was detected in 0.8% of MP isolates during 2006–2016 [37].

A very low prevalence of MR-MP was reported in Sweden. During the 1996–2017 period, only 0.2% of identified MP strains showed macrolide resistance [23]. However, a case of MR-MP infection after macrolide treatment was identified [38].

In Denmark, despite high macrolide use during the epidemic of 2010–2011, the reported level of macrolide resistance was 1–3% [39]. In addition, during a 3-year period in Switzerland (2011–2013), only 1/50 samples revealed high-level macrolide resistance [40].

In the Netherlands and Finland, no MR-MP strains were identified during the 1997–2008 or 2017–2018 epidemics, respectively [41,42].

**Figure 2:**
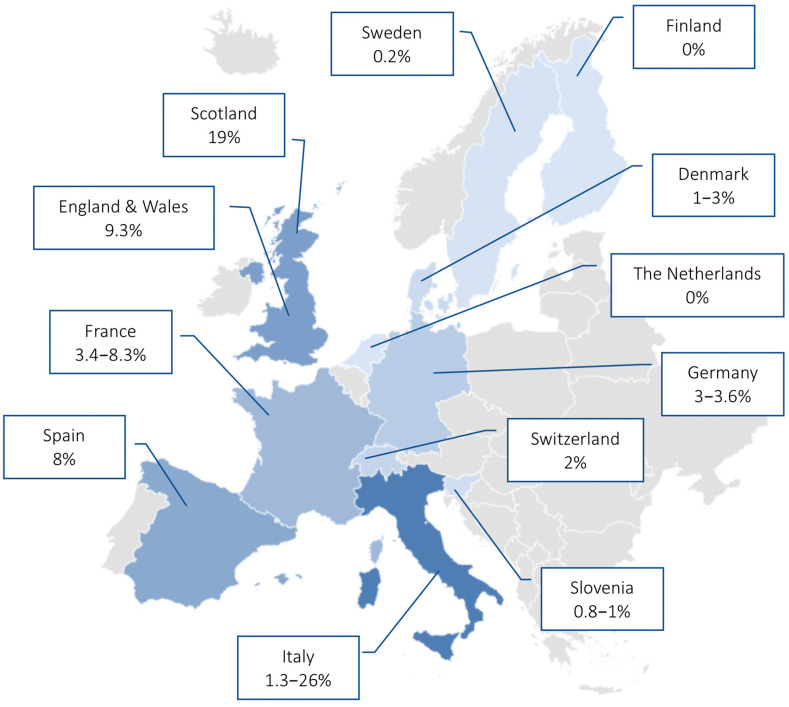
MR-MP prevalence rates reported in the European countries (years 1996–2019).

The MR-MP prevalence rates reported in the European countries are shown in Figure 2.

## 4. Discussion

This review provides an overview of the spread of MR-MP infection in Europe from 2010. The emergence of MR-MP strains is a public health concern since it represents a worrisome aspect of the global antimicrobial resistance threat. MR-MP strains have emerged since 2000, with rates of macrolide resistance increasing rapidly worldwide [43]. Resistance rates of 80–90% have been reported in Asia, particularly in China and Japan [43,44,45]. In recent years, however, there has been marked reduction in the incidence of MR-MP in Japan [46]. This reduction was registered after the 2011–2012 outbreak [47]; indeed, MR-MP rates dropped to 11.3% during the 2018–2019 period [48]. By contrast, high levels of MR-MP were maintained in China during the 2013–2018 period [49,50]. Compared with Asia, the spread of MR-MP in Europe is quite low. Most European countries lack national surveillance systems, resulting in an underestimation of the spread of MR-MP. This could represent a concern since no rapid alert system is available to identify an increase in MR-MP infections. The surveillance of MP across the European Union and European Economic Area countries is highly variable in terms of data collection and laboratory detection methods, thus making any comparison challenging [51]. The macrolide-resistant rates reported in Europe suggest that MR-MP strains lack a competitive advantage in a population moderately exposed to macrolides [27,52]. The highest prevalence was reported in Italy and Scotland during the 2010–2011 epidemic [16,34], while in the Netherlands and Finland, no MR-MP infections have been reported yet [41,42]. However, the sample sizes of the studies considered in this report were very different, so the prevalence rates should be carefully compared. 

The emergence of resistant strains of MP could be induced directly by macrolide use, even after a few days of therapy and particularly when a patient is exposed at suboptimal drug concentrations, as reported for other antibiotics [16,53]. However, it should be considered that a respiratory infection could be caused by a mixed population of susceptible and resistant strains, and that administration of a macrolide as therapy could drive selection of resistant species [22,54,55]. MP resistant rates could be influenced by the patient’s backgrounds and the epidemiologic situation in each country. For example, the reduction in MR-MP spread reported in recent years in Japan has been associated with the use of tosufloxacine rather than macrolides for the treatment of MP infections [13]. Moreover, the spread of the MP *p1* type may also explain this recovery of sensitivity to macrolides in Japan [13].

The usefulness of antibiotic treatment for MP infections is not clear since the majority of infections are self-limiting [56]. Macrolides appear to shorten the duration of symptoms; however, it is not clear whether this effect is due to their antimicrobial or anti-inflammatory properties [19]. In fact, the clinical relevance of MR-MP infections seems to be limited to prolonging symptoms rather than to increasing rates of complications [19], although one study reported that the incidence of extrapulmonary complications was higher in children presenting with MR-MP infection [57]. Replacing clarithromycin with levofloxacin, as reported in the study by Cardinale et al., led to prompt resolution of symptoms [19]. A longer duration of symptoms and hospitalization due to MR-MP infections should be considered as an alarm bell that triggers increased clinical vigilance. It should be considered that differences in MR-MP prevalence between out- and inpatients could be linked to higher rates of hospitalization in more severe cases, and in cases with a history of unsuccessful antibiotic treatment [27].

There is limited evidence regarding whether corticosteroids provide additional benefit for the treatment of MP infections [58]. The rationale underlying the addition of corticosteroids to antibiotics is that in cases with severe low respiratory tract infections caused by MP, the inflammation seems to be triggered by an excessive immune response rather than by the pathogen itself [59].

Atypical pneumoniae syndrome with fever, cough, and shortness of breath caused by MP could be difficult to differentiate from SARS-CoV-2 infection based on clinical presentation alone [60]. Those managing COVID-19 patients should bear in mind the possibility of the presence of other respiratory pathogens that cause co-infections. Co-infections of MP plus SARS-CoV-2 have been reported in the literature [61] and are associated with more severe disease characteristics [62]. Therefore, a SARS-CoV-2 diagnostic test should be undertaken, along with tests for other respiratory pathogens, to ensure better management of the patients. 

## 5. Conclusions

Monitoring the macrolide resistance of MP infection in Europe should be continued. Further studies based on whole-genome sequencing of MR-MP strains are needed to better understand the mechanisms underlying resistance and to investigate outbreaks. In addition, it is important to maintain low rates of infection. Moreover, a coordinated and structured surveillance program across Europe is required, both as a public health measure and to contain the spread of antimicrobial resistance.

## Figures and Tables

**Table 1:** Studies of the macrolide-resistant *Mycoplasma pneumoniae* (MR-MP) in Europe (2010–2021).

N.	Authors	Year of Study	Journal	Country	Type of Study	Population (Number of MP Positive Samples)	Diagnostic Methods for MP Identification	Main Findings
1	Dumke et al. [25]	2013	Antimicrobial Agents and Chemotherapy	Germany	Prospective study of out- and inpatients	Children and adults (n = 84)	Real-time PCR	Prevalence of MR-MP was 3.6% during an epidemic period
2	Dumke et al. [26]	2015	Emerging Infectious Diseases	Germany	Prospective study	Adults (n = 96)	Real-time PCR	Low levels of macrolide resistance (3.1%) during 2011–2012 period
3	Dumke et al. [27]	2019	Antimicrobial Agents and Chemotherapy	Germany	Retrospective study	Children and adults (n = 166)	Real-time PCR	Low rate of MR-MP infection (3%) during the 2016–2018 period
4	Dumke et al. [28]		International Journal of Infectious Diseases	Germany	Case report	15-year-old boy	Real-time PCR	Development of macrolide resistance after macrolide treatment. Presence of a mixture of wild-type and macrolide-resistant strains
5	Chironna et al. [16]	2011	Journal of Antimicrobial Chemotherapy	Italy	Prospective study of hospitalized patients	Children (n = 43)	Real-time PCR	MR-MP prevalence of 26% in children hospitalized for LRTI
6	Cardinale et al. [19]	2013	Journal of Clinical Microbiology	Italy	Prospective study of hospitalized patients	Children (n = 46)	Real-time PCR	The presence of MR-MP does not change the clinical presentation of CAP; however, children with MR-MP infection were more likely to show longer duration of fever, cough, and hospitalization
7	Chironna et al. [22]	2016	Medicine	Italy	Outbreak investigation	Children and adults (n = 8)	Real-time PCR	High transmission rates of MR-MP infection/carriage within a single family
8	Loconsole et al. [29]	2019	BioMed Research International	Italy	Retrospective study	Adults (n = 15)	Real-time PCR	1.3% of adults with CAP had a MR-MP infection
9	Pereyre et al. [30]	2012	PLOS One	France	Retrospective study	Children and adults (n = 34)	Real-time PCR	MR-MP prevalence of 3.4% in the 2007–2010 endemic period
10	Pereyre et al. [31]	2013	Clinical Microbiology and Infection	France	Retrospective study in hospitalized patients	Children and adults (n = 94)	Real-time PCR, culture, serology	MR-MP prevalence of 8.3% in the 2011 epidemic period
11	Caballero et al. [32]	2014	Antimicrobial Agents and Chemotherapy	Spain	Case report	23-year-old female	Real-time PCR, culture, serology	First description of MR-MP in a Chinese 23-year-old female coming back from China 13 days before CAP onset
12	Rivaya et al. [33]	2020	Journal of Antimicrobial Chemotherapy	Spain	Retrospective study	Children and adults (n = 138)	Real-time PCR, serology	MR-MP detected in 8% of infections in the 2013–2017 period
13	Ferguson et al. [34]	2013	Journal of Medical Microbiology	Scotland	Retrospective study	Children and adults (n = 32)	Real-time PCR	MR-MP prevalence of 19% in the 2010–2011 period, which was associated with a significant burden on local hospital admissions
14	Brown et al. [35]	2015	Euro Surveillance	England and Wales	Retrospective study	Children and adults (n = 43)	qPCR	MR-MP detected in 9.3% of collected samples
15	Kogoy et al. [36]	2015	Euro Surveillance	Slovenia	Retrospective study in out- and inpatients	Children and adults (n = 1255)	Real-time PCR, culture	Sporadic identification by MR-MP in the 2006–2014 period covering two nationwide epidemics
16	Kogoy et al. [37]	2018	European Journal of Clinical Microbiology & Infectious Disease	Slovenia	Retrospective study in out- and inpatients	Children and adults (n = 1477)	qPCR, culture	0.8% of MP isolated in the 2006–2016 period showed macrolide resistance
17	Nilsson et al. [38]	2014	Scandinavian Journal of Infectious Diseases	Sweden	Prospective study	Children and adults (n = 38)	Real-time PCR	1/38 cases developed MR mutations during macrolide treatment
18	Gullsby et al. [23]	2019	Journal of Clinical Microbiology	Sweden	Retrospective study	Children and adults (n = 578)	Real-time PCR	Very low prevalence (0.2%) of macrolide resistance in the 1996–2017 period
19	Uldum et al. [39]	2012	Euro Surveillance	Denmark	Data from MP national surveillance system	Children and adults (n = 140)	Real-time PCR	Low rate of MR-MP infections (1–3%) during the three waves in the 2010–2011 epidemic
20	Meyer Sauteur et al. [40]	2014	Swiss Medical Weekly	Switzerland	Prospective study in out- and inpatients	Children (n = 50)	Real-time PCR	Low level of macrolide resistance (1/50) during a 3 year period (2011–2013)
21	Kurkela et al. [41]	2019	European Journal of Clinical Microbiology & Infectious Disease	Finland	Outbreak investigation	Children and adults (n = 535)	Real-time PCR, serology	No MR-MP infections identified in the 2017 outbreak in Kimenlaakso
22	Spuesens et al. [42]	2012	Journal of Clinical Microbiology	The Netherlands	Retrospective study	Children and adults (n = 96)	Nested-PCR	MR-MP genotypes not found in 114 MP positive samples collected during the 1997–2008 period

## Data Availability

Not applicable.
